# 2203: Engraftment and gene expression of an HIV resistant immune system in a Phase I trial of an HIV stem cell gene therapy strategy

**DOI:** 10.1017/cts.2017.120

**Published:** 2018-05-10

**Authors:** Joseph Anderson, Kyle Hendrix, Julie Beegle, Jan A. Nolta, Mehrdad Abedi

## Abstract

OBJECTIVES/SPECIFIC AIMS: To date, only 1 documented case of an individual cured of HIV has been reported. He received an allogeneic bone marrow transplant with cells harboring an HIV-resistant genotype. To mimic this result, we have initiated a Phase I to evaluate the safety of an autologous stem cell gene therapy bone marrow transplant in HIV-related lymphoma patients. METHODS/STUDY POPULATION: The first cohort of patients will receive a 1:1 ratio of unmanipulated CD34 hematopoietic stem cells (HSC) and lentivector modified CD34 HSC expressing a combination of HIV-resistant genes and a selectable marker for cell sorting prior to transplantation. Safety of the HIV-resistant stem cells will be assessed by evaluating engraftment, expression of the anti-HIV genes, and the stability and sequence of the vector. RESULTS/ANTICIPATED RESULTS: One patient has been enrolled and transplanted with the HIV-resistant stem cells. After 1 and 2 months post-transplant, patient blood samples were received, processed for genomic DNA, analyzed by quantitative PCR (qPCR), and displayed successful engraftment of 16 and 12 vector copies per 100 cells, respectively. Expression of all anti-HIV genes was confirmed by qPCR. PCR on genomic DNA confirmed the correct sizes and sequences of the integrated vector and confirmed the successful engraftment of our gene modified cells. Currently, we are enrolling more patients into the trial. DISCUSSION/SIGNIFICANCE OF IMPACT: If successful, this therapy has the potential to change HIV treatment.

